# Personality Traits in Patients with Neuroepithelial Tumors – A Prospective Study

**DOI:** 10.1038/s41598-018-34980-w

**Published:** 2018-11-19

**Authors:** Jens Gempt, Stefanie Bette, Jennifer Albertshauser, Jasmin Hernandez Cammardella, Corinna Gradtke, Benedikt Wiestler, Lucas Schirmer, Yu-Mi Ryang, Bernhard Meyer, Florian Ringel

**Affiliations:** 1 Neurochirurgische Klinik und Poliklinik, Klinikum rechts der Isar, Technische Universität München, Ismaninger Str. 22, 81675 München, Germany; 2 Abteilung für Neuroradiologie, Klinikum rechts der Isar, Technische Universität München, Ismaninger Str. 22, 81675 München, Germany; 3 Department of Neurology, Klinikum rechts der Isar, Technical University of Munich, Ismaninger Str. 22, 81675 Munich, Germany; 4 Department of Neurology, University Medical Center Mannheim, University of Heidelberg, Theodor-Kutzer-Ufer 1-3, Mannheim, 68167 Germany; 5 Neurochirurgische Klinik und Poliklinik, Johannes Gutenberg-Universität Mainz, Langenbeckstraße, Mainz, 155131 Germany

## Abstract

Aim of this study was to analyze personality traits in patients with neuroepithelial brain tumors. Personality alteration is a common feature in brain tumor patients, but not much is known about associations between specific personality changes and brain tumors. We assessed potential factors influencing personality such as tumor location, tumor grade and tumor volume. Mini-mental state examination (MMSE), Beck’s Depression Inventory II (BDI-II), and the NEO Five-Factor Inventory (NEO-FFI) for the five factors of personality were acquired. Patients had lower scores regarding the factor openness and higher scores regarding the BDI-II compared to the norm population. No significant influencing factors (tumor entity, location) were found regarding personality traits. Neuroticism was associated with depression, whereas extraversion showed an opposed association. Patients with intrinsic brain tumors have differences in personality traits compared to the control population, with an emphasis on the factor openness. No significant confounding factors like tumor grade, entity, or location were found for personality traits.

## Introduction

In modern medicine, personality changes due to brain damage have been known for a long time. One of the first modern reports of personality changes is the one of Phineas Gage, a railroad construction foreman who survived a penetrating wound and damage to the prefrontal cortex by an iron rod. His relatives and friends noted major changes in his personality as consequence of the severe accident^1^. Patients with brain tumors often report of changes in personality or are concerned about potential changes after brain surgery or other treatment of the disease. Today, very little is known about the influence of brain tumors on personality. Most of the former oncological studies dealing with patients with primary brain tumors conducted assessments using the Karnofsky performance status scale (KPS) only, and for the assessment of higher cognitive functions, motor function or speech tests^2–5^. Nowadays though cognitive testing is more and more included into neurooncological studies and its importance is recognized^6–8^.

The development of personality during life is divided in three columns: human as an actor (behaving), agent (striving), and author (narrating)^9^. Today, the five-factor model (FFM) of personality is a widely accepted reference tool that is used in numerous studies of psychiatric and personality disorders^10^. The FFM enables the study of clinical and general personality traits alike. Factors included in the model are openness, conscientiousness, extraversion, agreeableness, and neuroticism and are summarized under the acronym OCEAN. The FFM has proven itself to be a robust model, which provides a simple outline of personality. Previous studies also showed that personality traits are closely related to depressive symptoms, e.g. a positive correlation between depression and neuroticism and a negative correlation between depression and extraversion^11,12^. Many studies addressed personality in neurological diseases e.g. dementia and stroke.

Alzheimer’s disease was shown to be associated with high neuroticism and low openness/extraversion and conscientiousness; these personality traits are also discussed as risk factors for dementia^13–15^. The hypothesis that especially neuroticism might be a risk factor for incident Alzheimer’s disease is supported by a previous study that showed no personality changes in the preclinical phase of the disease^15^. Studies assessed personality traits after stroke and the relationship between personality and well-being after stroke^16,17^. Neuroticism was associated with decreased psychological well-being after stroke^16^. Studies about personality changes after stroke are rare; a recent study showed that carers of stroke patients perceived personality changes after stroke, a study by Jokela *et al*. reported a decrease in extraversion, openness and conscientiousness, but not in agreeableness after diseases like heart disease, cancer and stroke^18^.

Since there are no prospective studies focusing on personality traits and the correlation between personality traits and depression in patients with intrinsic brain tumors, the aim of this study was to outline factors that influence the personalities of patients with neuroepithelial tumors. We hypothesize that (i) brain tumor patients show different personality traits compared to the control population, (ii) factors like tumor volume and location influence personality traits (iii) personality traits change after brain tumor surgery and (iiii) brain tumor patients show different correlations between depression and personality traits.

## Material and Methods

### Patients

Adult patients with neuroepithelial tumors who received tumor resection or biopsy at the local Department of Neurosurgery between 11/2009 and 12/2012 were included. Data were assessed in a prospective database. Exclusion criteria were severe psychiatric disorders, inability to complete the respective questionnaires independently (therefore patients with an MMSE score of <19 were excluded from the study and from further data assessment (i.e. NEO-FFI, BDI-II)), absent consent to participate in the present study, necessity for an emergency/rapid surgical treatment, and contraindications for MRI. Additional extended neuropsychological data was assessed as well and is reported elsewhere^19^.

The present study has been approved by the local ethics committee and has been performed in accordance with the ethical standards laid down in the 1964 Declaration of Helsinki and its later amendments (Clinical Trial Registration Number: 2840/10). All patients included in the study signed the informed consent form. The characteristics of patients’ personalities and diseases were recorded.

Tumor entity, tumor volume, pre-treatment, and tumor entity were derived from the medical charts. Tumor location, tumor volume, and Karnofsky performance status scale (KPS) were assessed separately for the study. Tumor volume was assessed in T2 weighted (w) fluid attenuated inversion recovery (FLAIR) using MRI sequences and T1 contrast enhanced sequences with semiautomatic segmentation (Iplannet 3.0, Brainlab AG, Munich).

### Outcome measures

Patients underwent personality evaluation according to the NEO Five-Factor Inventory (NEO-FFI) by Costa and McCrae^20^. The results of the NEO-FFI are the respective sum scores of the factors openness, conscientiousness, extraversion, agreeableness, and neuroticism. The score ranges from 0 to 48. For every factor, a population matched mean score is available. High-test results in the different factors are not recognized as a disease or necessarily disease specific but are characteristics of the respective personality.

Additionally, depression was assessed by the Beck’s Depression Inventory II (BDI-II)^21^. Basic cognitive function was assessed by the mini-mental state examination (MMSE), patients with a score <19 were excluded^22^. The described data were assessed preoperatively (t_0_), early postoperatively (t_1_), and 3 months (t_2_) and 9 months (t_3_) postoperatively. The MMSE was measured by a member of the research team, the questionnaires NEO-FFI and BDI-II were completed by the patient under supervision of the research team.

The BDI-II includes 21 items resulting in a score (range: 0 to 63) and is considered a reliable tool in the diagnostic of depression^23^.

The MMSE has 11 items and a maximal score of 30. A low score indicates cognitive deficits, the score of the normal population is stated with 27.6^24^.

### Statistical data analysis

Statistical analyses, including descriptive data analyses, were performed using IBM SPSS Statistics versions 22.0 and 23.0 (IBM Corporation, New York). Data was analyzed regarding normal distribution (histograms, QQ-plots, Kolmogorov-Smirnov- and Shapiro-Wilk-test). Normally distributed data are shown as mean and standard deviation. Non-normally distributed data are shown as median and interquartile range (IQR) and were analyzed with Spearman’s rank correlation coefficient. Comparisons between pre- and postoperative tests were analyzed with the Wilcoxon-test for paired samples. For all analyses, a difference with an error probability of less than 0.05 was considered to be statistically significant. Bonferroni-correction for multiple testing was performed.

## Results

### Clinical data

197 patients were invited to participate in the study, 112 patients agreed to participate. Of these 112 patients 39 patients were excluded due to histopathological result different from intrinsic brain tumor, withdrawal of consent or MMSE below 19.

Altogether, 73 patients (36 female, mean patient age at t_0_ 49 years, range 18 to 81 years, SD 17.6) were included. Median preoperative tumor volume was 16.3 cm^3^ (1.9–38.8) in T2w FLAIR images and 1.0 cm^3^ (0.0–22.3) in post contrast T1w images. Median postoperative tumor volume was 1.0 cm^3^ (0.0–9.9) in T2w FLAIR and 0.0 cm^3^ (0.0–0.4) in T1w images after contrast. Regarding the MMSE in our patients we observed a median score of 28 (IQR 26–29) at t_0_ and 29 (27–29) at t_1_.

At t_0_ (6.7 d preoperatively, SD 11.6), NEO-FFI was performed for all included patients. At t_1_ (mean = 11 d postoperatively, SD 11), 45 patients completed the NEO-FFI. At t_2_ (mean = 111 d postoperatively, SD 27.9), NEO-FFI was completed by 24 patients. Finally, at t_3_ (mean = 300 d postoperatively, SD 98.7), NEO-FFI was completed by 13 patients. Due to the high attrition rate during follow-up examinations at time points t_2_ and t_3_ were not included for further analysis. To assess whether personality traits are related to attrition, comparisons between patients with further tests after t_1_ and without further tests after t_1_ were performed using the Mann-Whitney U Test. No significant differences were shown between the two groups for pre- and postoperative personality traits and BDI-II (Supplemental Fig. 1).

Baseline patient and tumor characteristics are shown in Table 1.

**Table 1:** Patient and tumor characteristics at different time points (non-normally distributed data shown as median (interquartile range); DNET: dysembryoplastic neuroepithelial tumor).

	t_0_	t_1_
n	73	45
age	48.8y (−/+17.6)	43.1y (−/+16.1)
sex, female	36/73 (49.3%)	20/45 (44.4%)
WHO grade I	17/73 (23.3%)	12/45 (26.7%)
- pineocytoma	10/17	7/12
- pilocytic astrocytoma	1/17	1/12
- ganglioglioma	3/17	2/12
- DNET	2/17	2/12
WHO grade II	14/73 (19.2%)	10/45 (22.2%)
- diffuse astrocytoma	8/14	6/10
- oligoastrocytoma	2/14	2/10
- oligodendroglioma	3/14	2/10
- neurocytoma	1/14	—
WHO grade III	10/73 (13.7%)	8/45 (17.8%)
- anapl. Astrocytoma	6/10	4/8
- anapl. Oligoastrocytoma	3/10	3/8
- anapl. oligodendroglioma	1/10	1/8
WHO grade IV	32/73 (43.8%)	15/45 (33.3%)
- glioblastoma	31/32	15/15
- medulloblastoma	1/32	—
**Preoperative tumor volume**		
- T2 FLAIR (cm^3^)	16.3 (1.9–38.8)	13.9 (1.6–39.1)
- T1 contrast (cm^3^)	1.0 (0.0–22.3)	0.6 (0.0–10.5)
**Postoperative tumor volume**		
- T2 FLAIR (cm^3^)	1.0 (0.0–9.9)	0.1 (0.0–5.1)
- T1 contrast (cm^3^)	0.0 (0.0–0.4)	0.0 (0.0–0.0)
**Main tumor location**		
- frontal	21/73	11/45
- parietal	2/73	1/45
- temporal	14/73	9/45
- infratentorial	3/73	1/45
- midline	9/73	7/45
- multilobular without midline	17/73	15/45
- multilobular with midline	4/73	1/45
- ventricle	3/73	—
**Location, hemisphere**		
- right	30/73	22/45
- left	25/73	15/45
- midline	12/73	7/45
- both	6/73	1/45
**Personality traits**		
- neuroticism	20.0 (15.0–24.0)	20.0 (13.0–23.5)
- extraversion	28.0 (23.0–33.0)	29.0 (23.5–33.5)
- openness	27.0 (24.0–32.5)	26.0 (22.5–32.5)
- agreeableness	31.0 (29.0–35.0)	31.0 (27.5–35.0)
- conscientiousness	34.0 (30.5–37.0)	31.0 (28.5–38.5)
**Depression**		
- BDI-II	8.0 (5.0–13.5)	7.0 (4.0–10.0)

### BDI-II

**Figure 1:**
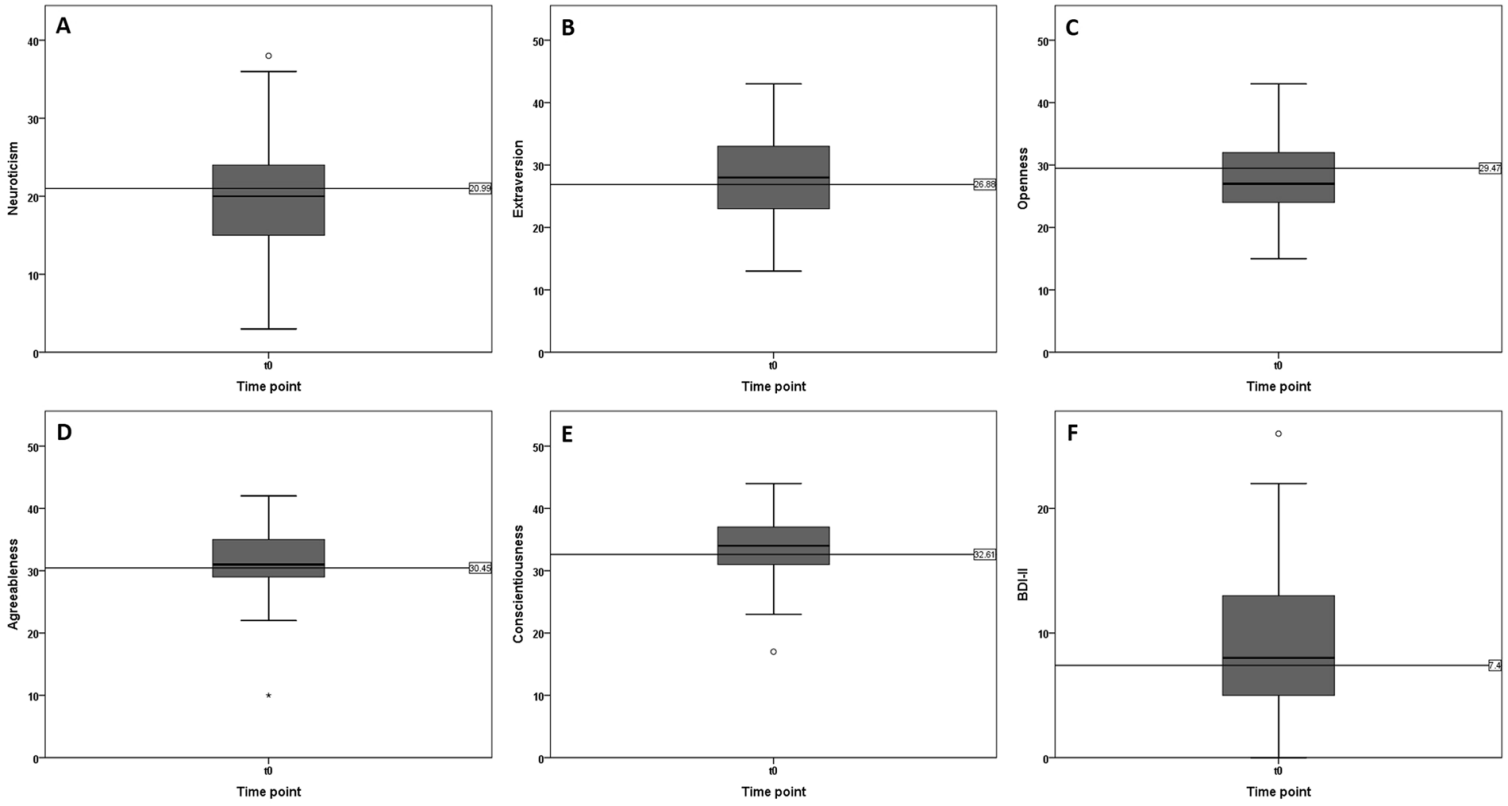
Mean values and standard deviation of scores for the five personality traits Neuroticism (**A**), Extraversion (**B**), Openness (**C**), Agreeableness (**D**), and Conscientiousness (**E**) compared to the mean of the normal population (-----).

The median value of the BDI-II in our patients was higher than that of the normative population regarding the level of depression. The reference value of the normative population was 7.4 (n = 582 depressive patients, n = 260 healthy controls, age ≥ 13 years, manual of Hautziger *et al*.^25^). The scores of our patients were 8.0 (IQR 5.0–13.5) at t_0_ (Fig. 1F). No significant associations between BDI-II and WHO-grade, tumor location (lobe/hemisphere) and tumor volume were observed (Table 2).

**Table 2:** Factors associated with personality traits: Neuroticism (A), Extraversion (B), Openness (C), Agreeableness (D), and Conscientiousness (E).

Feature	test	t_0_ (n = 73)
***A Neuroticism***		
WHO grade	Kruskal-Wallis	n.s.
Hemisphere	Kruskal-Wallis	n.s.
Tumor entity	Kruskal-Wallis	n.s.
Tumor location	Kruskal-Wallis	n.s.
Tumor volume (T1 contrast)	Spearman	n.s.
		r = 0.072
Tumor volume (T2 FLAIR)	Spearman	n.s.
		r = −0.023
BDI-II	Spearman	*P* = 0.0047
		r = 0.496
***B Extraversion***		
WHO grade	Kruskal-Wallis	n.s.
Hemisphere	Kruskal-Wallis	n.s.
Tumor entity	Kruskal-Wallis	n.s.
Tumor location	Kruskal-Wallis	n.s.
Tumor volume (T1 contrast)	Spearman	n.s.
		r = 0.072
Tumor volume (T2 FLAIR)	Spearman	n.s.
		r = −0.023
BDI-II	Spearman	*P* = 0.0047
		r = −0.413
***C Openness***		
WHO grade	Kruskal-Wallis	n.s.
Hemisphere	Kruskal-Wallis	n.s.
Tumor entity	Kruskal-Wallis	n.s.
Tumor location	Kruskal-Wallis	n.s.
Tumor volume (T1 contrast)	Spearman	n.s.
		r = 0.072
Tumor volume (T2 FLAIR)	Spearman	n.s.
		r = −0.023
BDI-II	Spearman	n.s.
		r = −0.071
***D Agreeableness***		
WHO grade	Kruskal-Wallis	n.s.
Hemisphere	Kruskal-Wallis	n.s.
Tumor entity	Kruskal-Wallis	n.s.
Tumor location	Kruskal-Wallis	n.s.
Tumor volume (T1 contrast)	Spearman	n.s.
		r = 0.072
Tumor volume (T2 FLAIR)	Spearman	n.s.
		r = −0.023
BDI-II	Spearman	n.s.
		r = −0.120
***E Conscientiousness***		
WHO grade	Kruskal-Wallis	n.s.
Hemisphere	Kruskal-Wallis	n.s.
Tumor entity	Kruskal-Wallis	n.s.
Tumor location	Kruskal-Wallis	n.s.
Tumor volume (T1 contrast)	Spearman	n.s.
		r = 0.072
Tumor volume (T2 FLAIR)	Spearman	n.s.
		r = −0.023
BDI-II	Spearman	n.s.
		r = −0.297
***F BDI-II***		
WHO grade	Kruskal-Wallis	n.s.
Hemisphere	Kruskal-Wallis	n.s.
Tumor entity	Kruskal-Wallis	n.s.
Tumor location	Kruskal-Wallis	n.s.
Tumor volume (T1 contrast)	Spearman	n.s.
		r = −0.079
Tumor volume (T2 FLAIR)	Spearman	n.s.
		r = −0.107

### NEO-FFI

The results for the five factors are given in Fig. 1(A–E) and in Table 2. Notable here is the low score regarding the factor openness at all tested time points compared to the average population (sample size n = 871, 423 male, 448 female, age distribution according to information of the German Federal Statistical Office, manual of Borkenau and Ostendorf 2008^26^) (Fig. 1C). The influence of WHO-grade, tumor volume, size, and location proved not to be significant according to mean values (Table 2).

### NEO-FFI and depression

**Figure 2:**
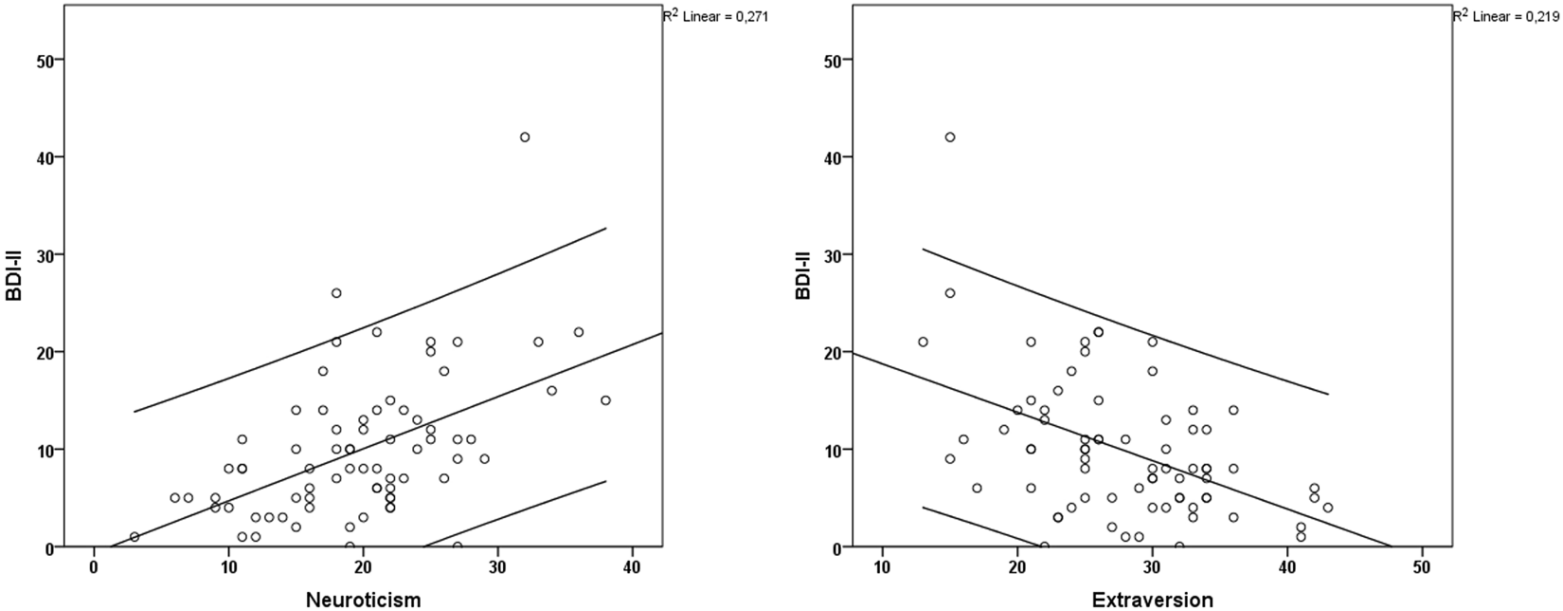
Correlation between BDI-II and the personality traits neuroticism and extraversion.

For depression (as measured with the BDI-II score) and neuroticism, a strong positive correlation was observed (t_0_, r = 0.496, *P* = 0.0047_;_ Fig. 2). In contrast, for depression and extraversion, a strong negative correlation was noted (from r = −0.413, *P* = 0.0047 Fig. 2).

### Pre- and postoperative comparisons

To analyze whether personality traits change after tumor resection, comparisons between time points t_0_ and t_1_ were performed. No significant changes were recorded for all analyzed personality traits (neuroticism: *P* = 0.565, extraversion: *P* = 0.799, openness: *P* = 0.060, agreeableness: *P* = 0.238 and conscientiousness: *P* = 0.328). Regarding descriptive statistics, a decline for the personality trait conscientiousness was observed (t_0_ median 34.0 [IQR 30.5–37.0] vs. t_1_ median 31.0 [28.5–38.5] which did not show statistical significance. BDI-II did also not significantly change pre- and postoperatively (*P* = 0.143).

## Discussion

This study investigated personality traits and depression in patients with intrinsic brain tumors. In contrast to the normal population, brain tumor patients had lower values for the personality trait openness before surgical resection and higher values regarding depression. No significant confounding factors for personality such as tumor grade, location, or entity were observed. Extraversion was inversely associated with depression, whereas neuroticism showed a positive correlation with depression. Personality traits remained stable after surgery.

To our knowledge this is the first study to investigate the subject of personality traits of patients with neuroepithelial tumors as a primary research object in a prospective manner. That changes of personality traits in brain tumor patients are existent and its potential impact is more and more discussed though^27^.

The values for openness were lower than those of the normal population. The other analyzed personality traits like extraversion and neuroticism were comparable to the normal population. All personality traits showed constant values over time, even despite brain surgery. This suggests that the personality of this patient cohort is very stable. However, this also raises the question as to why no personality changes were observed despite the reported mental changes in brain tumor patients^28^.

Changes in personality were recorded after stroke suggesting that alterations in brain tissue can affect personality traits. Elevated neuroticism and reduced extraversion were reported after stroke, and neuroticism was associated with reduced well-being^16,17^. Another study showed that personality traits changed after chronic diseases like heart disease, cancer and stroke: a decrease in extraversion, conscientiousness and openness was observed in this study^18^. Our study also showed a decline in conscientiousness, that however missed statistical significance. Comparisons of these studies is difficult as the type of the disease, the patient cohort and the time period differ a lot. The mentioned study by Jokela *et al*. assessed more than 17,000 patients over some years with different diseases including also heart disease and cancer, this study assessed only brain tumor patients pre- and postoperatively.

Personality changes may occur earlier in patients with brain tumors, or perhaps the tests were done too late after diagnosis in this cohort, but this is an inherent problem that cannot be resolved. Treatment (surgery, chemotherapy, or radiation) has less of an influence than the tumor itself. Personality changes may also occur later after surgery and/or diagnosis and were therefore not assessed in this study. Another explanation could be a selection bias in this study. Younger, healthier, and thus presumably more stable patients tended to participate in this study more than others. Furthermore, changes in personality traits might be very subtle and therefore not measurable with the NEO-FFI.

This study pointed toward a positive correlation between depression and neuroticism and a negative correlation between depression and extraversion in brain tumor patients. These results are in common with previous studies that showed correlations between these personality traits and depression, though not in brain tumor patients^11,29^. Another study analyzed a big cohort (n = 1980) and showed similar correlations between depression and personality traits, suggesting that brain tumor patients do not differ from the normal population with respect to these factors^12^.

No relevant correlations were observed in this cohort between personality traits and factors like tumor entity, location, or WHO grade.

A recent study showed that extraversion correlated with a decrease in grey matter in the frontal lobe and that agreeableness correlated with grey matter loss in posterior brain regions^30^. However, none of these personality traits showed correlations with tumor location in our cohort.

Regarding depression, all patients showed depression values that were worse compared to both the normal population and the previously published data by Rooney *et al*. for glioma patients^31^. In this study, however, a different test for depression was used. In common with another study, neither tumor entity and location, nor volume correlated with depression in our study cohort^32^.

Our study has some limitations. We have to consider the dropout-rate, a certain selection bias towards younger patients with a better cognitive function, which of course limits statement of the study. To address this high attrition rate, follow-up examinations were not analyzed in this study.

## Conclusion

In summary, patients with intrinsic brain tumors show differences in personality traits in contrast to the normal population, regarding the factor openness. A positive correlation between depression and neuroticism and a negative correlation between depression and extraversion were independently observed in brain tumor patients. Personality traits remained stable after tumor resection. No significant confounding factors like tumor grade, entity, or location were associated with personality changes in this circumscribed patient cohort.

## Electronic supplementary material

Supplemental Figure 1
