# Supplementary material for: Personality Traits in Patients with Neuroepithelial Tumors – A Prospective Study
Source: Sci Rep. 2018 Nov 19;8:17055. doi: 10.1038/s41598-018-34980-w (PMC6243000; doi:10.1038/s41598-018-34980-w)

Personality Traits in Patients with Neuroepithelial Tumors – A Prospective Study

Jens Gempt, MD[1], Stefanie Bette, MD[2], Jennifer Albertshauser[1], Jasmin Hernandez Cammardella[1], Corinna Gradtke[1], Benedikt Wiestler, MD[2], Lucas Schirmer[3,4], Yu-Mi Ryang, MD[1], Bernhard Meyer, MD[1], Florian Ringel, MD[1]

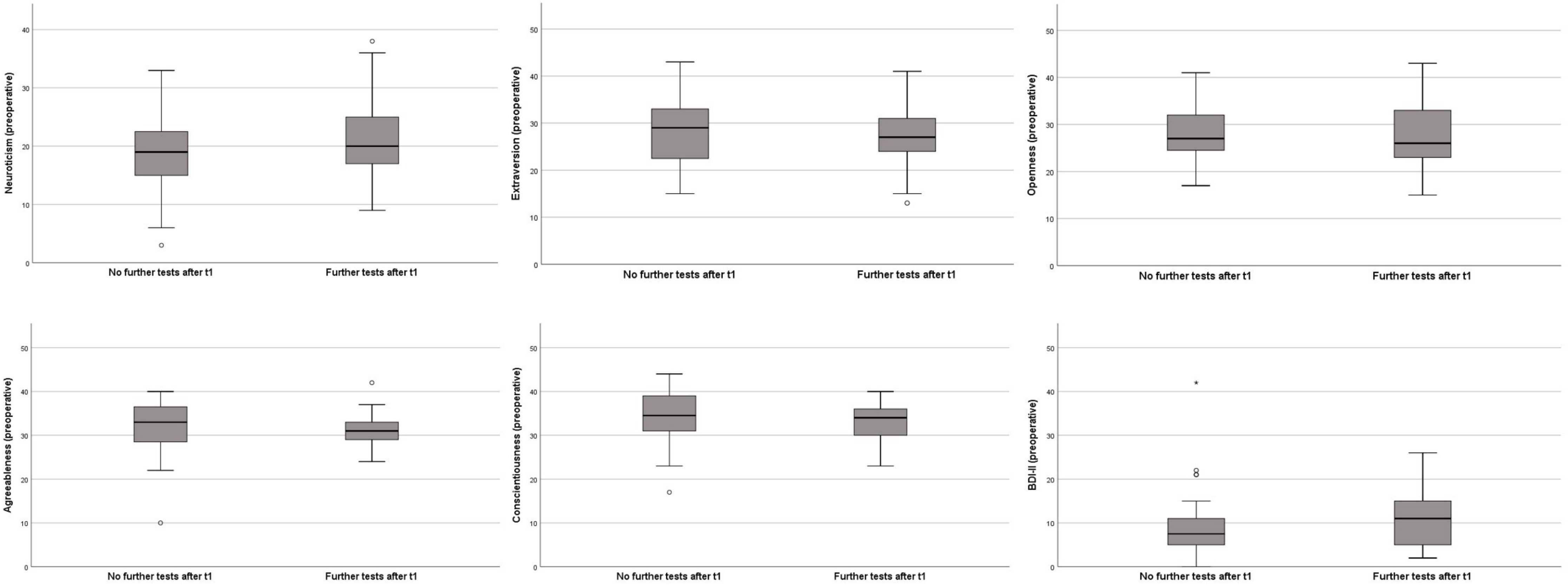

Supplement: Supplementary file 1 — Supplemental Figure 1 [file 41598_2018_34980_MOESM1_ESM.pdf]
